# A New Method for Precisely Designing the Spiral Structure of an SDD with Optimal Electrical Properties

**DOI:** 10.3390/mi17050585

**Published:** 2026-05-09

**Authors:** Xuyang Song, Jun Zhao, Tao Long, Chunxiang Ni, Xinqing Li, Manwen Liu, Xuran Zhu, Zhiyu Liu, Zheng Li

**Affiliations:** 1 School of Integrated Circuits, Ludong University, Yantai 264025, China; 16634270575@163.com (X.S.); longtao@ldu.edu.cn (T.L.); 13465506066@163.com (C.N.); 18663488310@163.com (X.Z.); 17861822572@163.com (Z.L.); 2 Engineering Research Center of Photo Detector Special Chip in Universities of Shandong, Ludong University, Yantai 264025, China; xqli1996zz@163.com; 3 School of Materials Science and Engineering, Xiangtan University, Xiangtan 411105, China; 4 Institute of Microelectronics, Chinese Academy of Sciences, Beijing 100029, China; liumanwen@ime.ac.cn

**Keywords:** Spiral Silicon Drift Detector, electric field distribution, electric potential profile, electron concentration distribution, full depletion voltage

## Abstract

To improve accuracy in calculating the radius of a spiral electrode and the number of turns in existing spiral-type Silicon Drift Detectors (SDDs), in this paper, we propose a method with which to derive first- and second-order approximations of differentiation equations for the spiral angle θ as a function of its radius r(θ) using Taylor expansion. Combining these with formulas for electrode pitch P(r) and width W(r), we developed a simple and physically intuitive method for obtaining a high-precision single-sided hexagonal spiral SDD. Comparisons of spiral structures calculated using the first- and second-order approximation formulas reveal that the second-order approximation yields more spiral turns, thus allowing superior electrical performance, including smoother electric potential profiles, more-uniform electric field distributions, and better-defined electron drift channels.

## 1. Introduction

Silicon Drift Detectors (SDDs) [1] demonstrate tremendous application potential across numerous fields, including material analysis [2,3,4], X-ray fluorescence spectroscopy [5,6,7], high-energy physics research [8,9,10], astrophysics [11,12], and medical imaging [13,14] (such as X-ray CT scans). SDDs are high-performance nuclear radiation detectors based on semiconductor physics mechanisms. Their innovative structural design significantly enhances particle detection accuracy and efficiency. Employing planar fabrication techniques, these devices feature specially patterned electrodes etched onto both surfaces of ultra-high-purity silicon wafers. Application of a lateral gradient bias voltage creates a uniform, strong electric field parallel to the detector surface within the depletion region. When charged particles or photons traverse this depletion layer, they lose energy and generate electron–hole pairs. The holes are rapidly captured by adjacent $p^{+}$-doped cathode electrodes, while electrons drift toward the collection anode region under the influence of the transverse drift electric field [15,16]. Efficient collection of these electrons yields precise information about the incident particles (energy, position, time, etc.).

In SDDs, n-type silicon wafers are typically used as the core substrate material. Their surfaces feature multiple drift ring structures formed by helical or concentric ring-shaped doping electrode layouts [17]. These detectors can be designed using geometric configurations, such as quadrilateral, hexagonal, or circular, to meet the physical requirements of different detection scenarios. In this paper, we primarily focus on hexagonal spiral-type SDDs. While recent advancements [18,19] in semiconductor detectors continuously push the boundaries of materials and structures, the proposed approximation methodology remains highly versatile and can be generalized to other geometries. For instance, applying this to circular SDDs yields optimal symmetrical electric fields ideal for single detectors, albeit with more dead space in arrays. Conversely, quadrilateral SDDs allow seamless array tiling at the cost of slight field non-uniformities at the corners. Compared with quadrilateral and circular SDDs, hexagonal SDDs provide the best compromise, facilitating easier array formation while reducing large dead zones. Compared with concentric ring-type SDDs, the spiral structure incorporates an automatic voltage-dividing function [20]. When bias voltages are applied to the innermost and outermost rings, a voltage gradient is automatically generated without the need for additional resistor networks for voltage division, which simplifies detector operation.

To improve calculation accuracy regarding the spiral ring radius and the number of turns in existing spiral SDDs, we propose a new, simple, and physically intuitive methodology for spiral SDD design. Unlike our previous preliminary studies, this work introduces an explicit analytical expression for θ(r) and a robust iterative scheme to solve the non-explicit r(θ) relationship (Table 1). Furthermore, we provide a quantitative analysis of the spiral turn difference η(R) to physically validate the performance enhancements. By employing this methodology together with optimized spiral pitch P(r) and width W(r) profiles, we derive a high-precision single-sided hexagonal spiral SDD structure with improved electrical properties.

## 2. Materials and Methods

To address the issue of computational accuracy, we propose a new calculation method by expanding θ(r+P(r)) around *r* using Taylor expansion:


(1) $$\theta \left(r+P\left(r\right)\right)=\theta \left(r\right)+\frac{1}{1!}\frac{d\theta }{dr}P\left(r\right)+\frac{1}{2!}\frac{d^{2}\theta }{dr^{2}}P^{2}\left(r\right)+\cdots$$

If we take the first-order approximation, we get

(2) $$\theta \left(r+P\left(r\right)\right)≅\theta \left(r\right)+P\left(r\right)\frac{d\theta }{dr}$$

When the radius *r* increases by a distance P(r), the angle θ increases by 2π, or the spiral completes one full turn:


(3) θ(r+P(r))−θ(r)=2π

We thus arrive at the differential equation using the first-order approximation:


(4) $$\frac{d\theta }{dr}=\frac{2\pi }{P(r)}$$

The first-order approximation yields a spiral design:


(5) $$\theta^{\left(1\right)}\left(r\right)=\int_{r_{1}}^{r}\frac{2\pi \;dr}{P(r)}$$

In Equation (5), which is the same as in ref. [17], $r_{1}$ is the initial (the first) radius of the helix; $P_{1}$ is the initial (the first) pitch of the helix; $\theta^{\left(1\right)}\left(r\right)$ is the polar angle obtained from the first-order approximation; and P(r) is the pitch of the helix.

To be more accurate, we now take the second-order approximation:


(6) $$\theta \left(r+P\left(r\right)\right)≅\theta \left(r\right)+P\left(r\right)\frac{d\theta }{dr}+\frac{1}{2}P^{2}\left(r\right)\frac{d^{2}\theta }{dr^{2}}$$

Again, when the radius *r* increases by a distance P(r), the angle θ increases by a full circle 2π (or the spiral completes one full turn). We now have:


(7) $$\frac{d\theta }{dr}=\frac{2\pi }{P(r)}-\frac{1}{2}P\left(r\right)\frac{d^{2}\theta }{dr^{2}}$$

We use the first order $\theta^{\left(1\right)}\left(r\right)$ in Equation (5) to calculate $\frac{d^{2}\theta }{dr^{2}}$:

(8) $$\frac{d^{2}\theta }{dr^{2}}=\frac{d}{dr}\left(\frac{2\pi }{P(r)}\right)=-\frac{2\pi }{P^{2}\left(r\right)}\frac{dP(r)}{dr}$$

By substituting Equation (8) into Equation (1), we acquire the second-order approximation $\theta^{\left(2\right)}\left(r\right)$:

(9) $$\theta^{\left(2\right)}\left(r\right)=\int_{r_{1}}^{r}\frac{2\pi }{P(r)}\;dr+\pi \int_{P_{1}}^{P(r)}\frac{dP(r)}{P(r)}$$

We now obtain the polar angle $\theta^{\left(2\right)}\left(r\right)$ from the second-order approximation:


(10) $$\theta^{\left(2\right)}\left(r\right)=\theta^{\left(1\right)}\left(r\right)+\pi \ln\frac{P(r)}{P_{1}}$$

It is clear that the second-order approximation solution $\theta^{\left(2\right)}\left(r\right)$ is a simple and clean expression in terms of the first-order approximation. Also, it is evident that the values of the second-order-approximation solutions in regard to the polar angle are larger than those of the first-order-approximation ones, and therefore there are more spiral turns.

To clearly observe the difference in the number of spiral turns between the first- and second-order approximations, we can calculate the following:

From Equation (10), we can find that


(11) $$\Delta \theta =\theta^{\left(2\right)}\left(r\right)-\theta^{\left(1\right)}\left(r\right)=\pi \ln\frac{P(r)}{P_{1}}$$

Therefore, the difference between the second- and first-order approximations is $\pi \ln\frac{P(r)}{P_{1}}$.

When Δθ=2π, it represents an increase of one full revolution (or full turn). Therefore, as *r* increases, Δθ increases. For any *r*, we define a number η(r) such that


(12) $$\Delta \theta \left(r\right)=\pi \ln\frac{P(r)}{P_{1}}=\eta \left(r\right)\cdot 2\pi$$

We then have

(13) $$\eta \left(r\right)=\frac{1}{2}\ln\frac{P(r)}{P_{1}}$$

which is the number of additional turns (at radius *r*) of the second-order approximation spiral relative to the first-order one.

Since the outermost radius of our spiral ring is *R*, the additional number of turns for our new design is as follows:

(14) $$\eta =\eta \left(R\right)=\frac{1}{2}\ln\frac{P(R)}{P_{1}}$$

In this work, we focus on the pitch function $P\left(r\right)=P_{1}{\left(r/r_{1}\right)}^{p}$, specifically choosing the exponent p=1/4. We chose p=1/4 because of the need to balance electric field continuity with microfabrication limits. Based on empirical modeling, the optimum range is 1/4≤p≤1/2. The value of 1/4 provides a pitch profile that is dense enough to ensure smooth and continuous electric field distribution, without making the pitch so small that it will cause lithographic-processing difficulties. By setting $r_{1}=100\;\mu m$, $P_{1}=50\;\mu m$, and R=5000μm, we arrive at

$$\eta \left(R\right)=\frac{1}{2}\ln\left(\frac{5000}{50}\right)=\frac{1}{2}\ln100=2$$

So, in this case, the second-order-approximation SDD spiral design has 2 more turns than the first-order one. Thus, it can provide more smooth and uniform distributions of electric potential and field as well as a well-defined electron drift channel.

In this work, we use the following form of the pitch function [17]:

(15) $$P\left(r\right)=P_{1}{\left(\frac{r}{r_{1}}\right)}^{\frac{1}{4}}$$

Assuming $r_{1}=100\;\mu m$, $P_{1}=120\;\mu m$, and R=2500μm, we obtain

$$\eta \left(R\right)=\frac{1}{2}\ln\left({\left(\frac{R}{r_{1}}\right)}^{\frac{1}{4}}\right)=\frac{1}{2}\ln\left({\left(\frac{2500}{100}\right)}^{\frac{1}{4}}\right)=\frac{1}{2}\ln\left(25^{\frac{1}{4}}\right)=0.4$$

Therefore, the number of turns obtained from the second-order-approximation solution in this case exceeds that of the first-order-approximation solution by 0.4 turns.

It should be noted that the increase in the number of spiral rings resulting from the second-order correction is marginal (e.g., an addition of fewer than 2 turns overall). This minor structural refinement does not increase lithographic complexity, nor does it noticeably elevate parasitic capacitance or resistance. Thus, manufacturability is fully preserved while electrical performance is enhanced.

By substituting P(r) from Equation (15) into Equation (5) and Equation (10), we obtain the first- and second-order-approximation expressions for the polar angles $\theta^{\left(1\right)}\left(r\right)$ and $\theta^{\left(2\right)}\left(r\right)$:

(16) $$\left\{\begin{array}{c} \theta^{\left(1\right)}\left(r\right)=\int_{r_{1}}^{r}\frac{2\pi \;dr}{P_{1}{\left(\frac{r}{r_{1}}\right)}^{\frac{1}{4}}}\\ \theta^{\left(2\right)}\left(r\right)=\int_{r_{1}}^{r}\frac{2\pi }{P(r)}\;dr+\pi \ln{\left(\frac{r}{r_{1}}\right)}^{\frac{1}{4}} \end{array}\right.$$

By completing the integrations in Equation (16), we finally obtain the spiral equations from the first- and second-order approximations:

(17) $$\left\{\begin{array}{c} \theta^{\left(1\right)}\left(r\right)=\frac{2\pi r_{1}}{P_{1}\left(1-\frac{1}{4}\right)}\left[{\left(\frac{r}{r_{1}}\right)}^{1-\frac{1}{4}}-1\right]\\ \theta^{\left(2\right)}\left(r\right)=\frac{2\pi r_{1}}{P_{1}\left(1-\frac{1}{4}\right)}\left[{\left(\frac{r}{r_{1}}\right)}^{1-\frac{1}{4}}-1\right]+\frac{1}{4}\pi \ln\frac{r}{r_{1}} \end{array}\right.$$

To make the actual spiral design more convenient, we need to determine the spiral radius as a function of the polar angle r(θ). For the first-order-approximation solution, one can easily derive from Equation (17) that

(18) $$r^{\left(1\right)}\left(\theta \right)=r_{1}{\left[1+\frac{\left(1-\frac{1}{4}\right)P_{1}\theta }{2\pi r_{1}}\right]}^{\frac{1}{1-\frac{1}{4}}}=r_{1}{\left[1+\frac{3P_{1}\theta }{8\pi r_{1}}\right]}^{\frac{4}{3}}$$

As for the second-order approximation solution in Equation (17), there is no explicit expression of *r* in term of θ. Instead, we may use the iteration method to obtain the approximated expression of $r^{\left(2\right)}\left(\theta \right)$.

First, we use $r^{\left(1\right)}\left(\theta \right)$ as the first iteration of $r^{\left(2\right)}\left(\theta \right)$—i.e., $r_{1}^{\left(2\right)}\left(\theta \right)=r^{\left(1\right)}\left(\theta \right)$; then, for the *n*th iteration, $r_{n}^{\left(2\right)}\left(\theta \right)$, we use

(19) $$\left\{\begin{array}{c} r_{n}^{\left(2\right)}\left(\theta \right)=r_{1}{\left[1+\frac{\left(1-\frac{1}{4}\right)P_{1}}{2\pi r_{1}}\left(\theta -\frac{\pi }{4}\ln\frac{r_{n-1}^{\left(2\right)}}{r_{1}}\right)\right]}^{\frac{4}{3}}\\ r_{1}^{\left(2\right)}\left(\theta \right)=r^{\left(1\right)}\left(\theta \right) \end{array}\right.\;\left(n=2,3,\ldots \right)$$

We will stop iteration until

(20) $$\frac{r_{n}^{\left(2\right)}\left(\theta \right)-r_{n-1}^{\left(2\right)}\left(\theta \right)}{r_{n}^{\left(2\right)}\left(\theta \right)}\leq 0.1\%$$

This 0.1% threshold was carefully selected to guarantee sufficient geometric precision for uniform electric field generation while remaining well within the physical resolution limits of standard silicon lithography. A looser threshold could introduce noticeable field non-uniformities.

In our case,

$$r_{2}^{\left(2\right)}\left(\theta \right)=r_{1}\cdot {\left[1+\frac{3P_{1}}{8\pi r_{1}}\left(\theta -\frac{\pi }{4}\ln\left[1+\frac{3P_{1}\theta^{\frac{4}{3}}}{8\pi r_{1}}\right]\right)\right]}^{\frac{4}{3}}$$

We stop at $r_{5}^{\left(2\right)}\left(\theta \right)$ when Equation (20) is satisfied.

We define the width of our spiral ring as follows:

(21) W(r)=αP(r)0<α<1

where α is the proportional coefficient relating *W* to *P*. In practical fabrication, α is limited by photolithography resolution. Assuming the minimum processable gap between spiral rings is 2μm, α must be ≤0.96 (for the minimum-pitch $P_{1}=50\;\mu m$ case). To balance field uniformity and prevent the formation of localized high-field regions near narrow gaps, a conservative optimal value of α=0.8 is utilized in our design. As P(r) changes continuously, W(r) also changes continuously in the same manner. Furthermore, to suppress leakage current during actual operation, the detector is designed to be cooled to ≤−30°C.

According to the formula for calculating the full depletion voltage [21],

(22) $$V_{fd}=\frac{qN_{eff}d^{2}}{2\varepsilon_{0}\varepsilon_{si}}$$

where *q* is the electron charge ($q=1.6\times 10^{-19}\;C$); $N_{eff}$ is the effective doping concentration of the silicon substrate ($N_{eff}=1\times 10^{12}\;{\mathrm{cm}}^{-3}$ in our work); *d* is the thickness of the silicon substrate (d=300μm); $\varepsilon_{0}$ is vacuum permittivity ($\varepsilon_{0}=8.854\times 10^{-12}\;F/m$); and $\varepsilon_{si}$ is the relative permittivity of silicon ($\varepsilon_{si}=11.9$). The full depletion voltage in this design is approximately 68.3V.

## 3. Structural Design and Electrical Performance Study

### 3.1. Structural Design of the Detector

A hexagonal shape was selected for the detector unit. Hexagons provide excellent symmetry close to an ideal circular geometry and facilitate array formation while minimizing dead zones.

**Figure 1:**
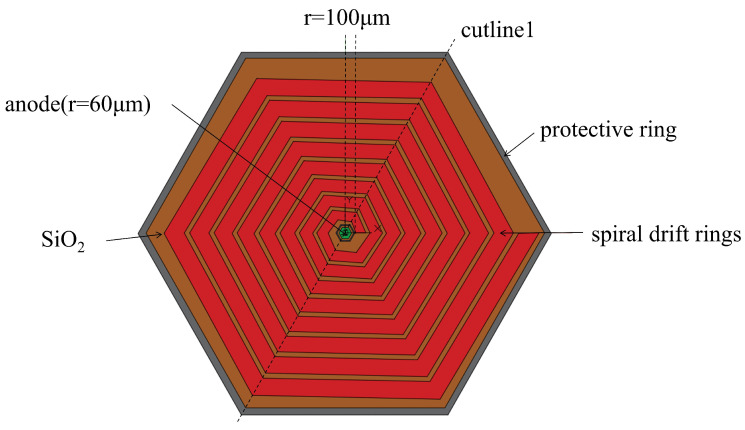
Front-side structural diagram of the detector.

The front-side structure of the detector (Figure 1) comprises (a) a central collection anode with a radius of 60μm, formed by an n-type region doped at a concentration of $1\times 10^{19}\;{\mathrm{cm}}^{-3}$ with a doping depth of 1μm; (b) a cathode ring—with a radius of 80μm and a width of 10μm—surrounding the anode; (c) spiral rings designed using the proposed methodology; and (d) a protective ring with a radius of 2470μm and a width of 50μm.

The innermost spiral ring has a radius of 100μm. The innermost cathode ring, spiral rings, and protective ring are heavily doped with $p^{+}$ impurities at a concentration of $1\times 10^{19}\;{\mathrm{cm}}^{-3}$ and a doping depth of 1μm. The cathode ring and protective ring help homogenize the surface electric field distribution and reduce leakage current effects.

The detector substrate consists of lightly doped n-type silicon with a concentration of $1\times 10^{12}\;{\mathrm{cm}}^{-3}$.

**Figure 2:**
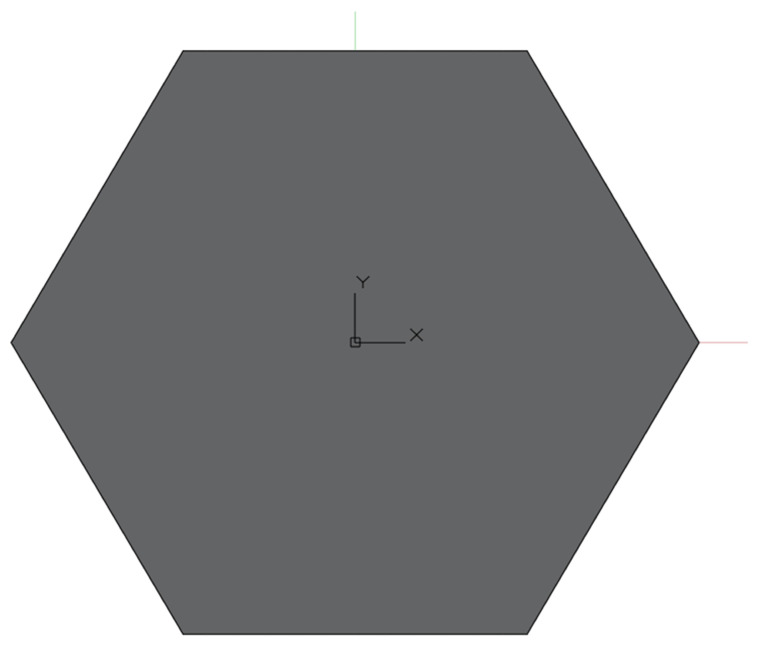
Backside structural diagram of the detector.

The backside structure of the detector, shown in Figure 2, comprises a full-surface cathode heavily doped with $p^{+}$ impurities at a concentration of $1\times 10^{19}\;{\mathrm{cm}}^{-3}$ and a doping depth of 1μm.

**Figure 3:**
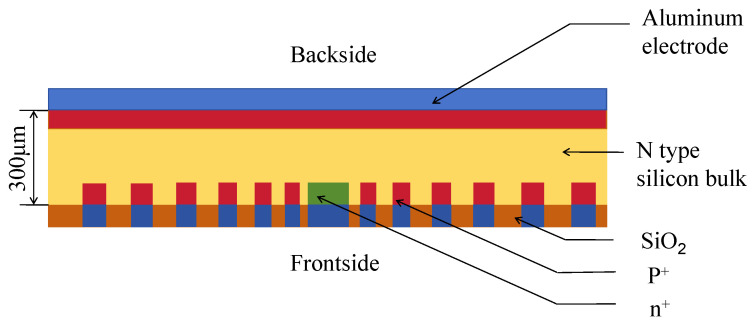
Cross-sectional structure of the detector.

Electrode contacts on the detector front (Figure 3) are defined at the anode, innermost cathode ring, the start and end points of the spiral ring, and the protective ring. The entire cathode on the rear surface has just one electrode contact (Figure 3). All areas with electrode contacts are covered with a 1μm thick aluminum layer. Areas without electrode contacts on both sides are covered with a 0.5μm thick ${\mathrm{SiO}}_{2}$ layer that was thermally grown before detector fabrication. It serves as both the passivation layer and the permanent mark for photolithography and ion implantation.

### 3.2. The Detector’s Electrical Properties

Sentaurus TCAD (Technology Computer-Aided Design) [22] is a professional software product for semiconductor device modeling and simulation. It employs numerical methods to simulate the physical structures and operational performance of detectors, thereby evaluating and optimizing their performance and reliability. This software product integrates multiple interconnected physics modules covering diverse physical processes, enabling collaborative simulation of key mechanisms such as carrier transport, electric field distribution, and recombination processes. It is widely applied in the high-precision modeling and design of semiconductor detectors. To further analyze the electrical properties and thus performance of the first-order and second-order structures of the spiral SDD, we simulated both structures using TCAD version 2018. To analyze the detector’s internal characteristics, we further elaborate on aspects such as the profile of the detector’s electric field, electric potential, and electron concentration below. To ensure the simulation was accurate, the Drift-Diffusion model, combined with Fermi–Dirac statistics, was employed to govern carrier transport. Recombination mechanisms, specifically Shockley–Read–Hall (SRH) and Auger recombination, were activated to reflect minority carrier lifetimes in high-purity silicon. Furthermore, a robust meshing strategy was implemented: a global coarse grid was applied to bulk silicon to optimize computational efficiency, while high-density local mesh refinement was executed at critical regions, notably the $p^{+}/n$ junctions and the surface of the device, to accurately resolve steep potential gradients.

#### 3.2.1. Electric Potential Distribution

**Figure 4:**
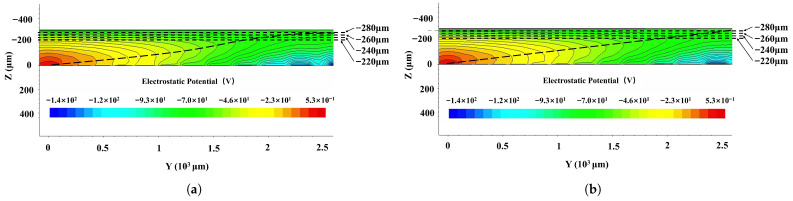
Electric potential distribution in SDD: (**a**) first-order and (**b**) second-order. Simulations were performed under bias conditions: $V_{anode}=0\;V$, $V_{cathode}=-2\;V$, $V_{E1}=-10\;V$, $V_{out}=-140\;V$, and $V_{B}=-70\;V$.

Figure 4 shows the electric potential profiles of the first- (Figure 4a) and second-order (Figure 4b)-approximation SDDs along the cross section shown in Figure 3 along cutline 1 in Figure 1. To clearly present the simulation results, in all figures for the simulation results, we only show half of the cross section shown in Figure 2 from r=0 to R=2470μm. Due to the detector’s planar symmetry, this does not affect our conclusions. The SDD biasing conditions are as follows [17]: (a) the anode is biased at 0 V, (b) the innermost cathode ring is biased at −2V, (c) the start of the spiral ring is biased at −10V ($V_{E1}=-10\;V$), (d) the end of the spiral ring is biased at −140V ($V_{out}=-140\;V$), and (e) the backside is biased at −70V ($V_{B}=-70\;V$).

As shown in Figure 4, the electric potential contours of both the first- and second-order-approximation SDDs are well defined and show clear paths toward the collection anode, as indicated by the arrow-marked lines. However, the electric potential contours and marked paths are clearer, smoother, and better defined for the second-order-approximation SDD.

**Figure 5:**
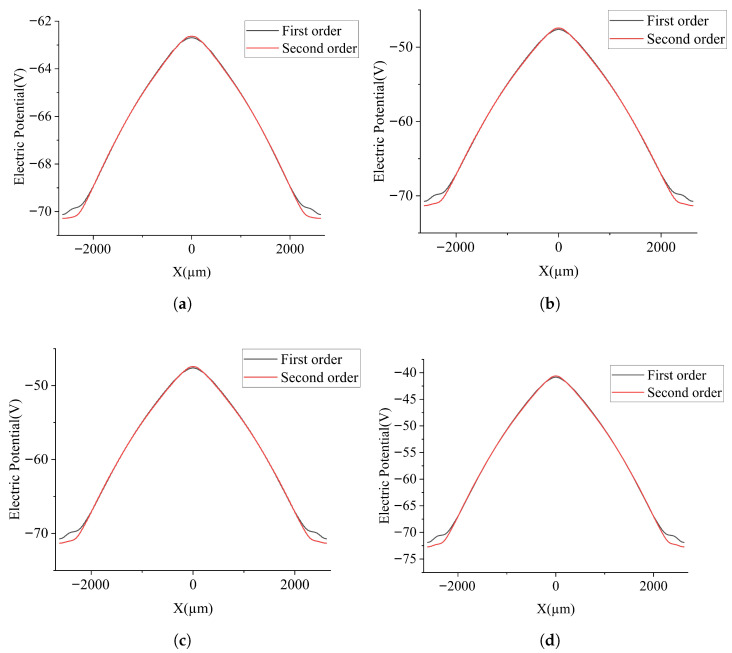
Distribution of potential in one-dimensional section of SDD: (**a**) Z=−280μm, (**b**) Z=−260μm, (**c**) Z=−240μm, and (**d**) Z=−220μm.

To further examine the differences between the two SDDs, additional cross sections at constant *Z* are analyzed in Figure 5. Figure 5a shows the one-dimensional electric potential distribution along the line Z=−280μm. The main differences between the two SDDs appear near r=0 and r=R. The electric potential is significantly smoother for the second-order-approximation SDD than for the first-order one. This observation is further confirmed by the electric potential profiles shown in Figure 5b–d at Z=−260μm, −240μm, and −220μm, respectively.

#### 3.2.2. Electric Field Distribution

**Figure 6:**
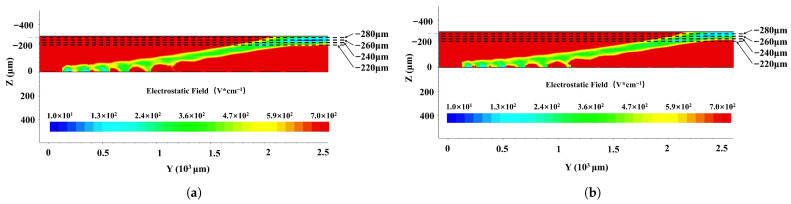
Electric field distribution in SDD: (**a**) first-order and (**b**) second-order. Simulations were performed under bias conditions: $V_{anode}=0\;V$, $V_{cathode}=-2\;V$, $V_{E1}=-10\;V$, $V_{out}=-140\;V$, and $V_{B}=-70\;V$.

Figure 6 shows the electric field profiles of the first- (Figure 6a) and second-order (Figure 6b)-approximation SDDs along the cross section shown in Figure 3 along cutline 1 in Figure 1. Both the first- and second-order-approximation SDDs are all fully depleted in both detectors. In both detectors, there exist clear low-electric-field (*E*-field) regions that define the electron drift channels. The low electric field in the drift channels is mainly dominated by the lateral electric field component, which demonstrates a high uniformity of approximately 380±10V/cm. In contrast, the electric field in regions outside the drift channels exceeds 600V/cm and is mainly dominated by the vertical electric field component. It can be clearly observed that the electric field profile of the second-order-approximation SDD is smoother and better defined than that of the first-order one. Specifically, the field uniformity along the drift channel was recorded to be 380±10V/cm, ensuring stable carrier transport.

**Figure 7:**
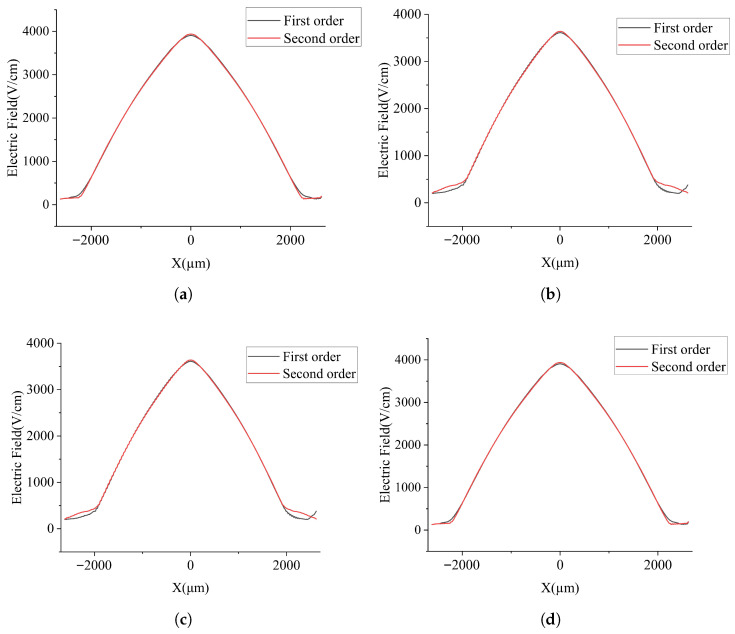
Distribution of electric field in one-dimensional section of SDD: (**a**) Z=−280μm, (**b**) Z=−260μm, (**c**) Z=−240μm, and (**d**) Z=−220μm.

To further examine the differences between the two SDDs, additional cross sections at constant *Z* will now be analyzed. Figure 7a shows the one-dimensional electric field profile along the line Z=−280μm. The main differences between the two SDDs appear near r=0. Near this region, the second-order-approximation SDD exhibits better symmetry than the first-order one. These differences are further confirmed by the electric field profiles shown in Figure 7b–d at Z=−260μm, −240μm, and −220μm, respectively.

#### 3.2.3. Electron Concentration Distribution

**Figure 8:**
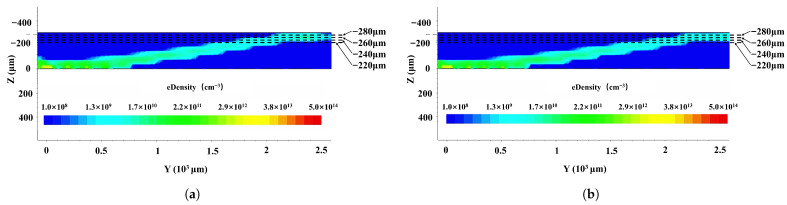
Electron concentration distribution in SDD: (**a**) first-order and (**b**) second-order. Simulations were performed under bias conditions: $V_{anode}=0\;V$, $V_{cathode}=-2\;V$, $V_{E1}=-10\;V$, $V_{out}=-140\;V$, and $V_{B}=-70\;V$.

Figure 8 shows the electron concentration profiles of the first- (Figure 8a) and second-order (Figure 8b)-approximation SDDs along the cross section shown in Figure 3 along cutline 1 in Figure 1. Electrons are clearly depleted throughout the detectors for both the first- and second-order-approximation SDDs, with electron concentrations below $10^{10}\;{\mathrm{cm}}^{-3}$, which is much smaller than the equilibrium concentration of $10^{12}\;{\mathrm{cm}}^{-3}$. Electron drift channels with lower electron concentrations toward the collection anode have clearly formed in both detectors. However, the electron drift channels in the second-order-approximation SDD are better defined, with narrower and smoother channel structures. Quantitatively, the highly uniform lateral electric field of approximately 380V/cm in the optimized drift channel ensures accelerated and complete charge transport. According to Equation (23), under these conditions, the maximum calculated electron drift time from the detector periphery to the anode is strictly contained to less than 0.5μs. Because the structural design does not negatively impact intrinsic anode capacitance or induce radiation trapping, this rapid drift time provides a strong theoretical indication that highly efficient charge collection and excellent energy resolution can be expected.

(23) $$t_{\mathrm{dr}}=\frac{L}{\mu E}$$

where *L* is the large drift length, μ is electron drift mobility ($\mu =1450\;{\mathrm{cm}}^{2}/V\cdot s$), and *E* is the drift field (380V/cm).

## 4. Conclusions

This paper proposes a more precise spiral SDD calculation and design methodology that is simple and physically intuitive. Our computations show that the second-order-approximation structure achieves higher accuracy than the first-order one, resulting in a larger number of spiral turns. Our electrical simulation results further demonstrate that the second-order-approximation SDD exhibits superior electrical properties compared with the first-order one. The main conclusions are that the second-order-approximation SDD exhibits (a) clearer, smoother, and better-defined electric potential contours and drift paths; (b) smoother and better-defined electric field profiles; and (c) better-defined electron drift channels with narrower and smoother channel structures. This new calculation and design methodology can provide useful guidance for future SDD device design and fabrication.

## Figures and Tables

**Table 1:** Geometrical parameters of the hexagonal spiral cathode rings.

Turn	Angle θ	1st-Order $r^{\left(1\right)}\left(\theta \right)\;\left(\mu m\right)$	2nd-Order $r^{\left(2\right)}\left(\theta \right)\;\left(\mu m\right)$
0	0	100	100
1	2π	235.33	219.56
2	4π	394.65	365.89
3	6π	572.27	532.16
4	8π	765.03	714.69
5	10π	970.85	911.13
…	…	…	…

## Data Availability

The original contributions presented in the study are included in the article. Further inquiries can be directed to the corresponding authors.
